# Epigenetic rewiring of skeletal muscle enhancers after exercise training supports a role in whole-body function and human health

**DOI:** 10.1016/j.molmet.2021.101290

**Published:** 2021-07-10

**Authors:** Kristine Williams, Germán D. Carrasquilla, Lars Roed Ingerslev, Mette Yde Hochreuter, Svenja Hansson, Nicolas J. Pillon, Ida Donkin, Soetkin Versteyhe, Juleen R. Zierath, Tuomas O. Kilpeläinen, Romain Barrès

**Affiliations:** 1 Novo Nordisk Foundation Center for Basic Metabolic Research, Faculty of Health and Medical Sciences, University of Copenhagen, Blegdamsvej 3B, 2200, Copenhagen N, Denmark; 2 Department of Physiology and Pharmacology, Karolinska Institutet, 171 77, Stockholm, Sweden; 3 Department of Molecular Medicine and Surgery, Karolinska University Hospital, 171 76, Stockholm, Sweden

**Keywords:** Exercise, Skeletal muscle, Enhancers, GWAS, Histone

## Abstract

**Objectives:**

Regular physical exercise improves health by reducing the risk of a plethora of chronic disorders. We hypothesized that endurance exercise training remodels the activity of gene enhancers in skeletal muscle and that this remodeling contributes to the beneficial effects of exercise on human health.

**Methods and results:**

By studying changes in histone modifications, we mapped the genome-wide positions and activities of enhancers in skeletal muscle biopsies collected from young sedentary men before and after 6 weeks of endurance exercise. We identified extensive remodeling of enhancer activities after exercise training, with a large subset of the remodeled enhancers located in the proximity of genes transcriptionally regulated after exercise. By overlapping the position of enhancers with genetic variants, we identified an enrichment of disease-associated genetic variants within the exercise-remodeled enhancers.

**Conclusion:**

Our data provide evidence of a functional link between epigenetic rewiring of enhancers to control their activity after exercise training and the modulation of disease risk in humans.

## Highlights

• Exercise training changes in skeletal muscle gene expression is enriched for secreted factors.
• The activity of skeletal muscle enhancers undergoes substantial remodeling after exercise training.
• Skeletal muscle enhancer activity and gene transcription are strongly associated.
• Exercise training-remodeled enhancer regions are enriched for GWAS SNPs associated with human traits and diseases.

## 1 Introduction

Regular physical activity decreases the risk of multiple common disorders such as cardiovascular disease [1,2], type 2 diabetes [3,4], cancer [5], and neurological conditions [[6], [7], [8]], along with the overall risk of mortality [[9], [10], [11]]. The beneficial effects of exercise training on human health are partially driven by adaptations of the skeletal muscle tissue. Exercise-induced adaptations include coordinated changes in the expression of genes controlling substrate usage and metabolic efficiency in skeletal muscle [12]. In addition to the adaptations that occur within skeletal muscle cells, exercise exerts systemic effects on whole-body homeostasis by triggering the release of soluble factors from the muscle that signal to distal tissues, such as brain, liver, and adipose tissue [13]. The mechanisms by which training-induced adaptations of skeletal muscle orchestrate positive effects at the whole-body level are poorly understood.

During the past two decades, genome-wide association studies (GWAS) have identified thousands of genetic variants associated with human complex traits and diseases. The vast majority of these variants are located in noncoding DNA regions [14] which overlap with gene-regulatory regions, particularly enhancers [[14], [15], [16]]. Enhancers are distal regulatory elements that are bound by multiple transcription factors and drive gene expression by forming physical interactions with promoters. Enhancers can be identified at a genome-wide level based on transcription factor binding clusters [17], mapping of accessible chromatin through DNase-Seq/FAIRE-Seq [18,19], sequencing of bi-directional enhancer RNA (eRNA) [20,21], or by mapping enhancer-associated histone modifications, including monomethylation of lysine 4 on histone 3 (H3K4me1) and acetylation of lysine 27 on histone 3 (H3K27ac) [[22], [23], [24]]. Combined, these techniques have identified more than 1.5 million enhancers across hundreds of human cell lines [25] and demonstrated that enhancer activity is highly dynamic in a cell type-specific and physiological context-dependent manner [[26], [27], [28]]. Therefore, mapping of enhancers in different tissues or physiological conditions can inform the mechanisms by which disease-associated genetic variants regulate phenotypic changes and predispose to disease.

Here, we hypothesized that endurance exercise training remodels the activity of gene enhancers in skeletal muscle and that this process contributes to the beneficial effect of exercise on human health. We present the first mapping of human skeletal muscle enhancers after endurance training and show that training-responsive enhancers are enriched for genetic variants associated with human diseases and complex traits.

## 2 Methods

### 2.1 Human subjects

The study was performed on 8 Caucasian males (mean ± SD age 23 ± 4). The study was approved by the Ethics Committee from the Capital Region of Denmark (reference H-1-2013-064) and informed consent was obtained from all participants in accordance with the Declaration of Helsinki II. Investigations resulting from the use of a subset of the collected biopsies and other biological samples have been reported elsewhere [29,30].

### 2.2 Exercise intervention and collection of biopsies

Before the exercise intervention, biopsies were collected (pretraining) from all participants and a VO_2_max test was performed. The endurance exercise program was performed five days a week; 60 min supervised spinning classes for 6 weeks. The spinning classes were performed at 70% of the participants’ individual reserve capacity of their max pulse. All participants completed all training sessions. After the last bout of exercise, participants rested for 4 days before delivering the post-training sample and performing a second VO_2_max test. Both the pre- and post-training biopsies were collected with a Bergström needle with suction under lidocaine local anesthesia in a fasted and resting state from the *vastus lateralis*. The muscle biopsies were immediately snap-frozen in liquid nitrogen and stored at −80 °C until further analysis.

### 2.3 Total RNA purification

Skeletal muscle tissue (20–30 mg) was used for the purification of total RNA from each biopsy. After lysis of the tissue in RLT buffer (Qiagen) using a Tissuelyzer II (Qiagen, 30 Hz for 3 × 30 s), total RNA was purified using AllPrep DNA/RNA/miRNA Universal Kit (Qiagen). The quality of recovered RNA was assessed by the Agilent RNA 6000 Nano Kit (Agilent Technologies), and RNA concentration was determined by spectrophotometry using a NanoDrop 2000 (Thermo Scientific).

### 2.4 RNA-sequencing

For RNA-sequencing, 0.5 μg of total RNA was depleted of rRNA and subsequently used to generate libraries using the TruSeq standard total RNA with Ribo-Zero Gold kit (Illumina). The PCR cycle number for each library amplification was optimized by running 10% of the library DNA in a real-time PCR reaction using Brilliant III Ultra-fast SYBR Green QPCR Master Mix (AH Diagnostic) and a C1000 Thermal cycler (Bio-Rad). Libraries were sequenced on a NextSeq500 system (Illumina) using the NextSeq 500/550 High Output v2 kit (75 cycles). Using the Kallisto aligner [31] v. 0.46, reads were aligned to the hg38 ENSEMBL release 79 transcripts [32], with a transcript support level between 1 and 3. Read summation onto genes and differential expression analysis was performed by Sleuth [33] v. 0.30. Differential expression *p*-values and Bonferroni corrected q-values were calculated using a likelihood ratio test, comparing a model containing only participant information (~participant) to a model with participant and training status information (~participant + training). Fold changes were calculated using a Wald test, as described in the Sleuth manual. Two samples (P6_2 and P8_2) were observed to express high levels of genes associated with the dermis, which was not observed in the other samples. These samples were excluded from differential expression analysis. Real-time quantitative PCR (RT-qPCR) validations were performed by using Brilliant III Ultra-fast SYBR Green QPCR Master Mix (AH Diagnostic) and a C1000 Thermal cycler (Bio-Rad). All reactions were analysed in quadruplicates. The following primer sequences were used: *COL4A2* (F:5′-TTCCTCATGCACACGGC-3′, R: 5′-TTCGATGAATGGTGTGGCG-3′), *COL5A1* (F:5′-AAGCGTGGGAAACTGCTCTC-3′, R: 5′-AGCCGCAGGAAGGTCATCTG-3′), *EMILIN1* (F:5′-GCCCCAAG-TGGCATTTTCAG-3′, R: 5′-CAAGTAGCGTCCAGCCAGTG-3′), *FSCN1* (F:5′-GCGCCTACAACATCAAAGACTC-3′, R: 5′-GAAGTCCACAGGAGTGTCGC-3′), *NID1* (F:5′-TGTCACTTGGGAATCCGTGG-3′, R: 5′-CTGGAACGTGTTTCTCTTGCC-3′), *RASSF2* (F:5′-ATGCAGGATGACAACGAACG-3′, R: 5′-CTTCAGAGTGGTTCCCTGAGC-3′), *SLC9C1* (F:5′-GCTATAAGAGACCTTGGGCTTTC-3′, R:5′-ACAGAGGTCATCAGACTTTCTCC-3′). Expression was normalized to *TBP* (F:5′-CCCGAAACGCCGAATATAATCC-3′, R: 5′- AATCAGTGCCGTGGTTCGTG-3′).

GO enrichment analysis of genes that were upregulated after exercise training was performed by the online tool GOrilla [34], using all identified genes as the background dataset.

Genes encoding secreted gene products were downloaded from UniProt's subcellular localization annotation database [35] (March 5, 2019), from the ExoCarta database [36] (March 5, 2019).

### 2.5 Chromatin IP-sequencing (ChIP-seq)

For the preparation of chromatin from human skeletal muscle biopsies before or after training (n = 8), frozen biopsies (20–40 mg) were thawed on ice and chopped into small pieces (between 1 and 3 mm^3^). The tissue was fixated in 0.5% formaldehyde in PBS for 7.5 min at room temperature followed by quenching with glycine (final concentration of 0.125 M). The fixated tissue was washed with PBS before resuspension in 1 ml of IP buffer (67 mM Tris–HCl (pH 8), 100 mM NaCl, 5 mM EDTA (pH 8.0), 0.2% NaN3, 0.33% SDS, 1.67% Triton X-100, 0.5 mM phenylmethylsulfonyl fluoride) and dounce homogenization was performed until the tissue was completely dissociated. Chromatin was sonicated (Diagenode, Biorupter) to an average length of 200–500 bp (between 20 and 30 cycles; high intensity).

Before starting the ChIP experiment, chromatin was cleared by centrifugation for 30 min at 20,000 g. For each ChIP, chromatin corresponding to 2 μg DNA was combined with a 2.5 μg antibody and incubated with rotation at 4 °C for 16 h. Immunoprecipitation was performed by incubation with Protein G Sepharose beads (GE healthcare) for 4 h followed by three washes with low-salt buffer (20 mM Tris–HCl (pH 8.0), 2 mM EDTA, 1% Triton X-100, 0.1% SDS, 150 mM NaCl) and two washes with high-salt buffer (20 mM Tris–HCl (pH 8.0), 2 mM EDTA, 1% Triton X-100, 0.1% SDS, 500 mM NaCl). Chromatin was de-crosslinked in 120 μl 1%SDS and 0.1 M NaHCO_3_ for 6 h at 65 °C, and DNA was subsequently purified using Qiagen MinElute PCR purification kit. For library preparation and sequencing, 2–7 ng of immunoprecipitated DNA was used to generate adaptor-ligated DNA libraries using the NEBNext Ultra DNA library kit for Illumina (New England Biolabs) and indexed multiplex primers for Illumina sequencing (New England Biolabs). The PCR cycle number for each library amplification was optimized by running 10% of the library DNA in a real-time PCR reaction using Brilliant III Ultra-fast SYBR Green QPCR Master Mix (AH Diagnostic) and a C1000 Thermal cycler (Bio-Rad). DNA libraries were sequenced on a HiSeq2000 by 50-bp single-end sequencing at the National High-Throughput Sequencing Centre (University of Copenhagen, Denmark). Reads were aligned to a full index of the main chromosomes of the hg38 reference genome, using the Subread aligner v1.5.0 [37], with the genomic DNA and unique only flags set. Picard tools (http://broadinstitute.github.io/picard) were used to remove duplicate reads. To assess the quality of the IP step, narrow peaks were called on each sample using the MACS2 peak caller [38] v2.1.0.20150731. Samples were flagged as low-quality if the fragment lengths could not be estimated, or if less than 200.000 peaks could be called with a *p*-value cut-off of 0.05. These peaks were only used for quality control and were not used in any downstream analysis. No samples were discarded. For each histone mark, the consensus peak sets used for testing differential binding were generated following the ENCODE 2012 IDR pipeline: All samples were pooled (pooled samples) and reads were randomly shuffled and split into two files (pseudo-replicates). Peaks were called on pooled samples and pseudo-replicates using the same parameters as for the individual samples. Finally, the consensus peak list was generated using the irreproducible discovery rate (IDR) software [39] v2.0.2, with a cut-off of 0.05. The peak lists from the pseudo-replicates were used as input and the peak lists from the pooled samples were used as oracle lists. The IDR is similar to the FDR, but has been shown to result in more reproducible peak sets than FDR cut-off defined lists [40]. Counts for the individual samples were summarized onto peaks using feature counts [41] v1.20.6. Differential binding was found using the quasi-likelihood functions of edgeR [42] as previously described [43], using all counts along the entire peak. The model used had the form ~ participant + training, where participant encoded participant ID and the training was 0 for the untrained state and 1 for the trained state. When selecting peaks with both H3K27ac and H3K4me1, peaks were considered overlapping if they overlapped by a single nucleotide.

MDS plots for both RNA and ChIP-seq experiments were generated by removing participant effects using the remove batch effect function, and subsequently, calculating MDS coordinates using plot MDS function; both of which are part of the edgeR package [42].

GO enrichment analysis of genes near enhancers that were activated after exercise training was performed by the online tool GREAT [44] using all identified enhancer regions as the background dataset. Before the analysis, peaks were lifted to the hg19 reference genome using the UCSC liftOver tool [45].

Motif enrichment was performed using MEME-ChIP [46] in “discriminative mode”. Enhancers were resized to a width of 500 bp centred on the middle of the peak. Peaks with an FDR less than 0.1 were used as a foreground, while enhancers with an FDR greater than 0.1 were used as control sequences.

Enhancers were connected to genes using EpiMap [47]. The per group muscle gene-enhancer links were downloaded from the EpiMap repository and lifted to hg38 using the UCSC LiftOver tool [45]. A single nucleotide overlap with the EpiMap enhancers was considered sufficient to connect an enhancer to the linked gene.

### 2.6 GWAS overlap

#### 2.6.1 Data retrieval from the GWAS catalog

The data retrieval and analysis process is depicted in Supplemental Figure S6. All SNPs associated with complex diseases or other traits were downloaded from the GWAS catalog v1.0.2 [48] (November 19, 2018) and SNPs not reaching the genome-wide significant *p*-value < 5 × 10^−8^ were excluded. We only included unique SNPs with information on genome position; haplotypes were excluded. LD clumping: To avoid bias caused by the presence of linkage disequilibrium (LD) between SNPs – which could lead to single genetic signals being accounted more than once – we applied LD clumping with an R^2^ threshold of 0.5 to all SNPs; such that only the SNP with the strongest *p*-value in each LD block was included in the final clumped list. The resulting clumped list was used in the disease/trait-specific and disease/trait-combined analyses, respectively.

#### 2.6.2 Categorization of GWAS catalog traits

We grouped the diseases and traits into 19 categories which all had a minimum of 150 disease/trait SNPs per category. The trait categories were grouped as follows: 1) anthropometric measurements, 2) autoimmune diseases, 3) bone mineral density, 4) cancer, 5) cardiovascular diseases, 6) cognitive-related, 7) coagulation, 8) inflammatory bowel disease, 9) inflammatory response, 10) lipid levels, 11) lung-related, 12) diabetes and glucose homeostasis, 13) neuropsychiatric, 14) obesity-related, 15) ophthalmological, 16) oxygen carriers, 17) renal function and diseases, 18) female reproductive traits, and 19) others. Supplemental Figure S7 gives details on how the categories were grouped using the “disease/trait” indicator from the GWAS catalog.

#### 2.6.3 Generation of control SNPs

Using the SNPsnap [49] tool, we generated a random set of control SNPs for comparison with the GWAS SNPs to estimate enrichment in enhancer regions. We used the SNPsnap recommended default matching settings: minor allele frequency of ±5%, gene density of ±50%, distance to the nearest gene of ±50%, and LD buddies ±50%, using R^2^ = 0.5. We requested two matched SNPs per each independent disease/trait SNP from the GWAS catalog-clumped SNPs. We then constructed a GWAS SNP vs. control SNP matching with a ratio of 1:2. For every disease/trait category, we excluded any control SNPs that were duplicates.

#### 2.6.4 Overlapping training-responsive enhancer regions and SNP positions

GWAS SNPs and control SNPs were overlapped with the positions of the 7018 enhancers that were differentially activated after long-term training using BEDTools [50] v2.25.0.

#### 2.6.5 Selecting tag SNPs across disease/trait categories

To avoid missing overlaps for SNPs in complete LD with the lead GWAS SNPs, we performed a matched tag SNP selection with an R^2^ threshold of 1 for each GWAS category and control SNPs separately. The contribution to the overlap of SNPs within the same LD block was considered as only one overlap; no matter how many SNPs in the LD block overlapped with the enhancer region. This step and LD pruning were performed with Plink v1.9 [51].

#### 2.6.6 Statistical analysis

We used logistic regression to test whether the GWAS SNP status was associated with higher odds of being in an enhancer region as compared to control SNPs in the 19 disease/trait categories separately and in combination. The overlap of the disease/trait SNPs and control SNPs in the enhancer regions was defined as 1 for the presence and 0 for the absence of overlap. The results are reported as ORs with error bars indicating 95% confidence intervals with the corresponding *p*-values. Statistical analyses were performed using STATA 15.

## 3 Results

### 3.1 Gene expression analysis identifies genes regulated after exercise training

**Figure 1:**
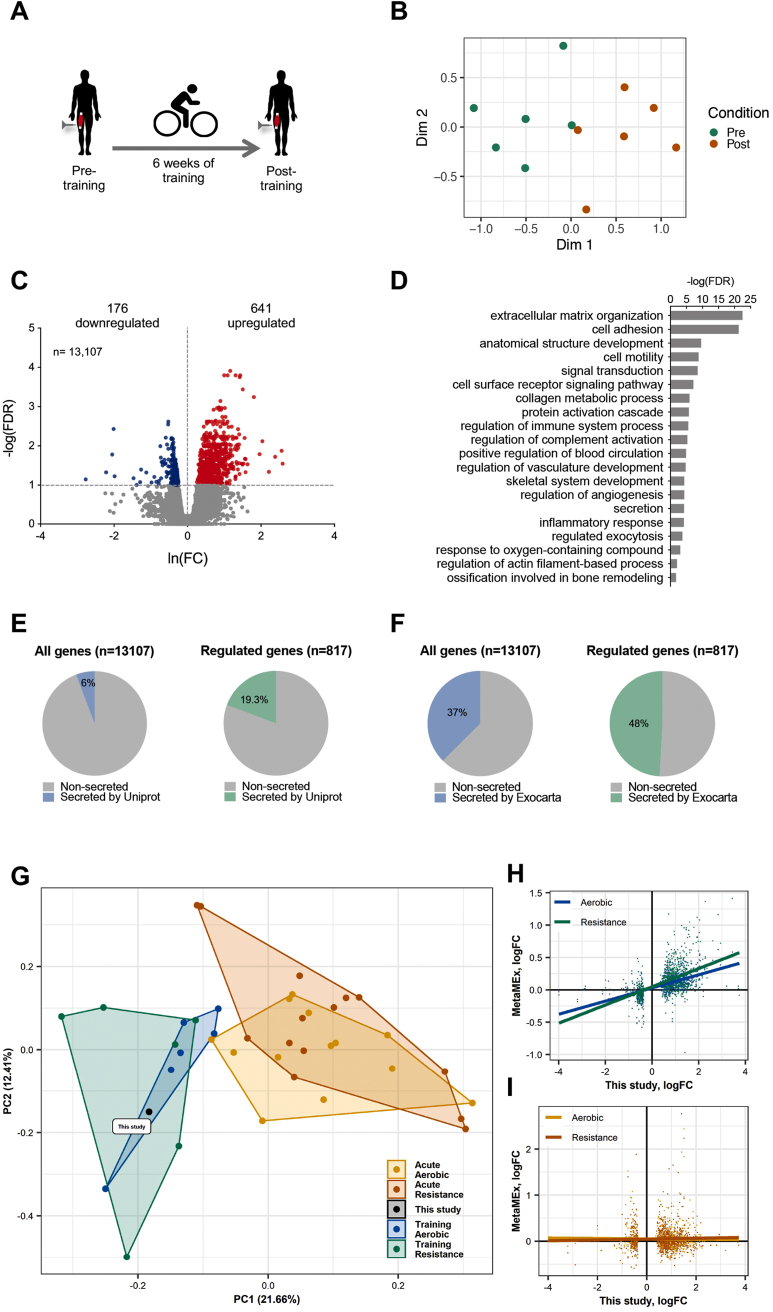
**Identification of genes regulated by exercise training in human skeletal muscle.** (**A**) Skeletal muscle biopsies were taken from the *vastus lateralis* of Caucasian males before (pre) or after (post) six weeks of endurance training consisting of supervised ergo cycle exercise for 60 min, five days a week, at 70% of each participant's maximal aerobic capacity. (**B**) MDS plot of RNA-seq data from human skeletal muscle biopsies taken before or after training. (**C**) Volcano plot representation of genes regulated by exercise training (n = 8 participants, FDR<0.1). (**D**) Selected gene ontology terms identified among genes upregulated after exercise training. (**E-F**) Fraction of all identified genes or genes regulated by exercise training that encode factors being annotated as secreted by Uniprot (E) or found secreted by exosomes, as annotated by the ExoCarta database (F). Genes encoding secreted factors are enriched within regulated genes for both Uniprot and ExoCarta (chi-square test, *p* < 1E-5). (**G),** All datasets of healthy individuals from the MetaMex meta-analysis and our dataset were compared with each other using a principal component analysis (**H–I).** Correlations between RNA-seq logFC of our identified differentially expressed genes and data from the MetaMex meta-analysis of aerobic (blue) or resistance (green) exercise training (H) or of acute aerobic (yellow) or resistance (orange) acute exercise (I).

We collected skeletal muscle biopsies from the *vastus lateralis* muscle of 8 sedentary Caucasian males before (pre training) and after (post training) a 6-week endurance training program consisting of supervised ergocycle exercise for 60 min, five days a week, at 70% of each participant's maximal aerobic capacity (Figure 1A) [29]. After completing the training program, we found that the average aerobic capacity, as measured by maximal oxygen uptake (VO_2_ max), was increased by 20% (Supplemental Figure S1). While body mass index (BMI) was not altered, the waist-to-hip ratio was decreased, implying that exercise training induced abdominal fat loss (Supplemental Figure S1).

We first investigated the effect of exercise training on the transcriptome of skeletal muscle by RNA sequencing (RNA-seq). A multidimensional scaling (MDS) plot of the RNA-seq data revealed a clear separation between pre- and post-training samples (Figure 1B). Among all 13,108 genes with a detected expression in the skeletal muscle biopsies (Figure 1C), we identified 641 genes upregulated in the trained state compared to the untrained state, whereas 176 genes were downregulated (false discovery rate (FDR) < 0.1) (Supplemental Data S1 and Figure 1C). From the upregulated genes, we selected 7 genes and performed RT-qPCR to validate the expression changes. Owing to the lack of biological materials, we excluded two participants from this analysis. Based on the remaining six individuals, we could still validate an upregulation of *COL5A1*, *EMILIN1*, *SLC9C1*, *FSCN1*, and *NID1*, whereas *COL4A2* and *RASSF2* tended to be upregulated (Supplemental Figure S2). Gene ontology (GO) analysis of the upregulated genes returned 421 enriched terms (Supplemental Data S2), including terms related to extracellular matrix organization, the immune system, angiogenesis, and actin filament sliding, along with terms related to exocytosis and the secretion of factors (Figure 1D). In accordance, when comparing exercise-regulated genes to all 13,108 skeletal muscle-expressed genes, we found an overrepresentation of genes encoding proteins annotated as *secreted* by UniProt's subcellular localization annotation [35] (Figure 1E), or as *secreted by exosomes* by the ExoCarta database [36] (Figure 1F). These genes encode extracellular matrix proteins, complement factors, chemokines, and cytokines, among others. When we compared the regulated genes encoding secreted factors with studies investigating the secretome of muscle cells [[52], [53], [54], [55]], we found that the majority of the proteins (65%) have been reported by at least one other group to be secreted from muscle (Supplemental Data S3), suggesting that many of the regulated genes are likely to encode proteins that serve as exercise-regulated myokines. To further validate our findings, we compared our transcriptomic data to a recent meta-analysis of gene transcription changes in human skeletal muscle after acute or long-term exercise training [56]. A subset of the MetaMEx database including only studies performed in healthy young males was used, and the meta-analysis included data sets from 6 studies of acute aerobic exercise (GSE59088, GSE71972, GSE87748, GSE107934, GSE120862, GSE33603), 8 studies of acute resistance exercise (GSE7286, GSE19062, GSE24235, GSE23697, GSE28422, GSE59088, GSE106865, GSE107934), 6 studies of aerobic-based exercise training (GSE111551, GSE120862, GSE139258, GSE24215, GSE35661, GSE9103), and 5 studies of resistance-based exercise training (GSE106865, GSE24235, GSE28422, GSE28998, GSE45426). A principal component analysis of gene expression responses in these studies shows a clustering of the data according to exercise intervention, where our dataset clustered with the aerobic and resistance training studies and not the acute exercise studies (Figure 1G). Accordingly, we found a positive correlation between logFC values of our differentially expressed genes and values from the meta-analysis with the aerobic and resistance training studies (Figure 1H), and not the acute exercise studies (Figure 1I). Collectively, these data show that exercise training improves metabolic and cardiorespiratory fitness and induces profound transcriptional changes in skeletal muscle, notably involving genes encoding secreted factors.

### 3.2 Remodeling of enhancer activities after exercise training

**Figure 2:**
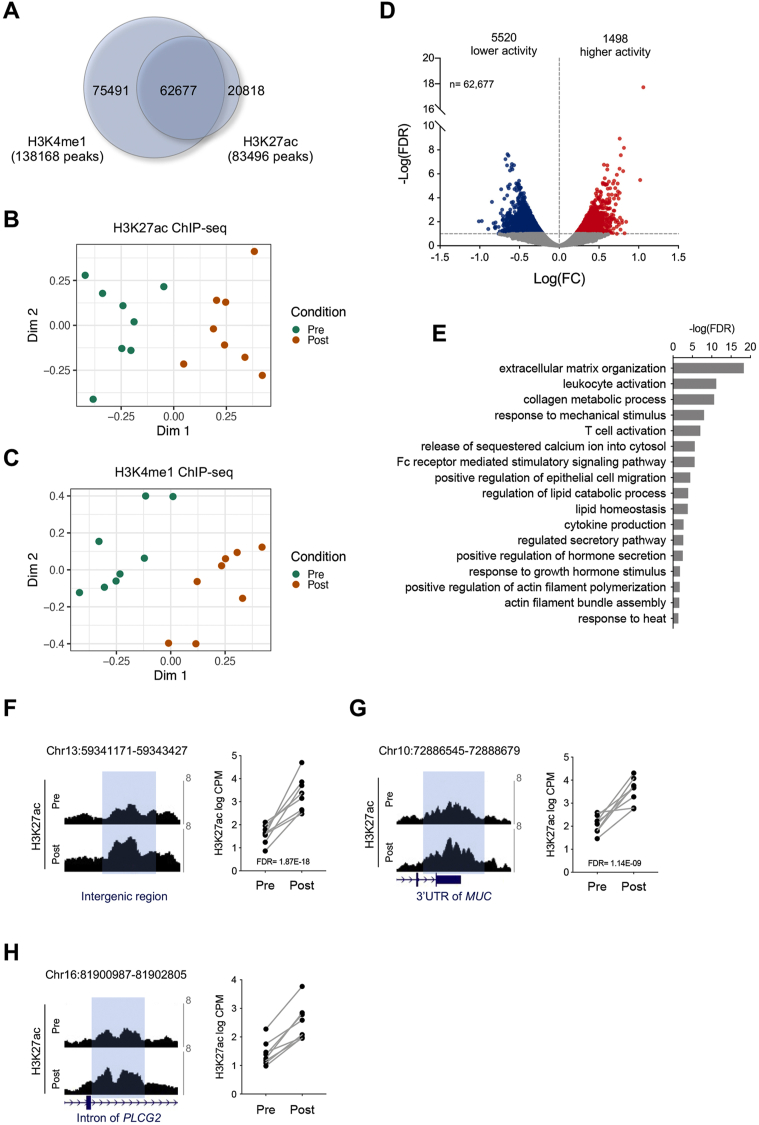
**H3K4me1 and H3K27ac ChIP-seq identify enhancers that are regulated after exercise training.** (**A**) Overlay of H3K4me1 and H3K27ac ChIP-seq peaks from human skeletal muscle biopsies. (**B–C**) Multidimensional scaling (MDS) plot of H3K27ac (B) and H3K4me1 (C) ChIP-seq data from muscle biopsies taken before or after training. (**D**) Volcano plot representation of differentially acetylated H3K27 regions among the 62,677 enhancers containing both H3K4me1 and H3K27ac (n = 8 participants, FDR<0.1). (**E**) Selected gene ontology terms identified by the bioinformatic tool GREAT of enhancers that become activated after exercise training. (**F–H**) The top three enhancers that change H3K27ac after exercise training. The figure shows UCSC genome browser (hg38) H3K27ac tracks (left panel) and quantification of H3K27ac counts per million (CPM) (right panel) from skeletal muscle biopsies taken pre- or post-exercise training (n = 8 participants).

To generate genome-wide maps of active enhancers in skeletal muscle, we performed chromatin immunoprecipitation (ChIP)-sequencing of DNA surrounding the modified histones H3K4me1 and H3K27ac. We identified 138,168 regions with significant enrichment of H3K4me1 and 83,496 regions enriched for H3K27ac in skeletal muscle (FDR<0.1). Most (75%) of the H3K27ac peaks were also covered by H3K4me1, whereas 45% of the H3K4me1 peaks showed enrichment of H3K27ac (Figure 2A). Consistent with our findings on gene expression, we found that MDS plots of ChIP-sequencing data showed a clear separation of samples based on the training status, both for ChIP-seq of H3K27ac (Figure 2B) and H3K4me1 (Figure 2C), indicating that exercise has a profound effect on the distribution of these histone marks. The levels of the H3K27ac mark are a prominent marker of enhancer activity. Thus, to identify enhancers differentially activated after exercise training, we searched for enhancers within the 62,677 regions covered by both H3K4me1 and H3K27ac, that showed significant changes in H3K27ac levels between the untrained and trained state. This analysis returned 7,018 enhancers with altered activity after exercise training, of which 5,520 had decreased and 1,498 had increased levels of H3K27ac (FDR<0.1) (Supplemental Data S4 and Figure 2D).

Next, we used the Genomic Regions Enrichment of Annotations Tool (GREAT) [44] to map the identified enhancers to neighboring genes, and thus to identify enriched gene ontologies. This analysis returned 69 enriched terms of which many were related to processes also found in the GO analysis of differentially expressed genes, such as extracellular matrix organization, immune-related processes, muscle contraction-related processes, and regulated secretion of factors (Supplemental Data S5 and Figure 2E). The top three enhancers that were regulated by exercise training were located on chromosome 13 (chr13:59341171–59343427) (Figure 2F), chromosome 10 (chr10:72886545–72888679) (Figure 2G), and chromosome 16 (chr16:81900987–81902805) (Figure 2H), as illustrated by the difference in the average H3K27ac signal between the pre- and post-training samples (left panel), and by the difference in H3K27ac counts pre- and post-training for each participant individually (right panel). We further performed motif enrichment analysis of the exercise-regulated enhancer regions using MEME-ChIP [46] and identified seven enriched motifs (Supplemental Figure S3A). When scanning these motifs for known transcription factor binding, we found evidence for the binding of FOXJ3, PRDM6, ANDR, ZN770, PAX5, and ZN121 transcription factors to some of the identified motifs, whereas other motifs had no known TF binding sites (Supplemental Figure S3B). Collectively, these results show that the activity of skeletal muscle enhancers undergoes substantial remodeling after exercise training, which might be driven by specific DNA motif sequences.

### 3.3 Training-responsive enhancers regulate the expression of connected genes

**Figure 3:**
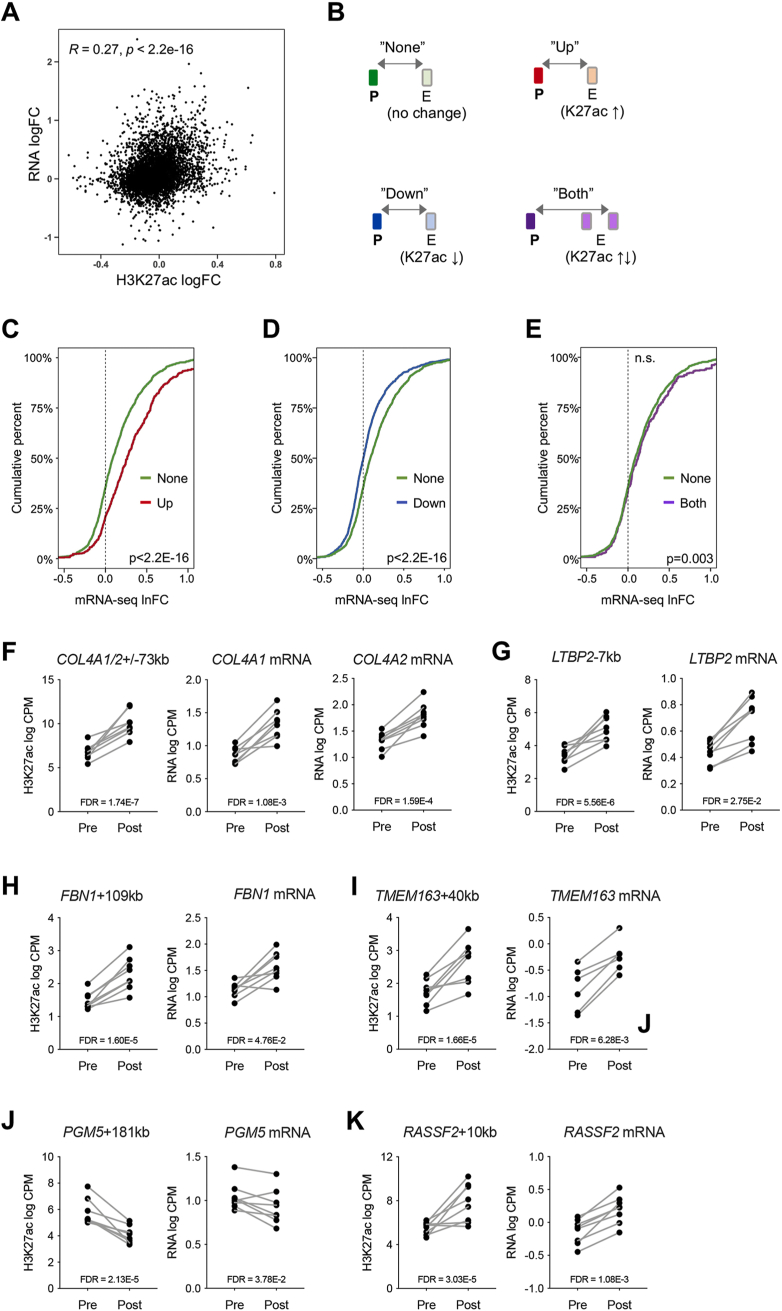
**Association between exercise-training responsive enhancers and expression of connected genes.** (**A**) Correlation between changes in gene transcription (RNA logFC) and enhancer activity (H3K27ac logFC). Enhancer-gene interactions were based on the strongest interactions identified by EpiMap. (**B**) Genes whose expression were detected in the RNA-seq analysis were divided into four groups; genes linked to enhancers showing no change in H3K27ac in response to exercise training (“None”), those linked to enhancers that either gained H3K27ac (“Up”) or lost H3K27ac (“Down”) in response to exercise training, and those linked to several enhancers, where some gained H3K27ac and others lost H3K27ac (“Both”). (**C-E**) Empirical cumulative distribution function (ECDF) plots of gene expression changes (RNA-seq lnFC values) in the “Up” versus the “None” group (C), the “Down” versus the “None” group (D), and the “Both” group versus the “None” group (E). The x-axis represents the RNA-seq lnFC and the y-axis is the fraction of genes with this lnFC or less. (**F–K**) The top six enhancer regions with changes in H3K27ac after training (left panel), where expression of a connected gene was also changing (identified from the RNA-seq analysis) (right panel).

To gain insight into the association between epigenetic remodeling of enhancers and gene expression changes in skeletal muscle in response to exercise training, we integrated recent data on skeletal muscle enhancer-gene links from EpiMap [47], which is based on transcriptomic and epigenomic analyses of 61 skeletal muscle samples. This allowed us to connect 36,038 of the identified muscle enhancers to one or more gene(s), with an average of 10.5 enhancers connected to each gene, and 3.3 genes per enhancer (Supplemental Figure S4A and B). We found that the average distance between the connected enhancers and genes was 239 kb (Supplemental Figure S4C). To investigate the correlation between the changes in enhancer activity and gene expression in response to exercise, we connected the identified enhancers to the gene with the highest interaction score and observed a positive correlation between the fold change of enhancer acetylation and gene transcription (Figure 3A). Furthermore, we divided promoters from our RNA-seq analysis into four groups: Promoters linked to an enhancer that did not change H3K27ac levels in response to exercise training (“None”), those linked to at least one enhancer that either gained (“Up”) or lost (“Down”) H3K27ac, and those linked to several enhancers, where some gained H3K27ac and some lost H3K27ac (“Both”) (Figure 3B). Empirical cumulative distribution function plots of gene expression changes (RNA-seq fold change (lnFC) values) in the four different groups revealed that promoters connected to enhancers with gained activity (“Up”) had higher lnFC values than the “None” group, whereas those connected to enhancers with decreased activity (“Down”) had lower lnFC values, thus supporting a regulatory role of the enhancers on transcription (Figure 3C,D). For promoters of the “Both” group, the gene expression changes were only slightly upregulated (Figure 3E). Similarly, we found that enhancers linked to either up- or down-regulated genes were also likely to gain or lose activity, respectively, compared to those linked to nondifferentially expressed genes (Supplemental Figure S5A–B).

Based on the integration of EpiMap enhancer-gene interaction, we identified 599 enhancer-gene interactions, covering 491 enhancers and 268 genes, where both the enhancer and the connected gene were either upregulated or downregulated after exercise training (Supplemental data S6). The top regulated enhancers – where we also detected transcriptional changes from a connected gene after exercise training – were elements connected to the shared promoter of *COL4A1* and *COL4A2* (*COL4A1/2* ± 73 kb, Figure 3F), and to the promoters of *LTBP2* (*LTBP2*-7 kb, Figure 3G), *FBN1* (*FBN1*+109 kb, Figure 3H), *TMEM163* (*TMEM163 + 40 kb*, Figure 3I)*, PGM5* (*PGM5*+181 kb, Figure 3J), and *RASSF2* (*RASSF2*+10 kb, Figure 3K)*.* Collectively, these analyses establish a strong association between changes in enhancer activity and gene transcription, which supports the functional role of enhancers in the cellular adaptations of exercise training.

### 3.4 Training-responsive enhancers are enriched for genetic variants identified by GWAS

**Figure 4:**
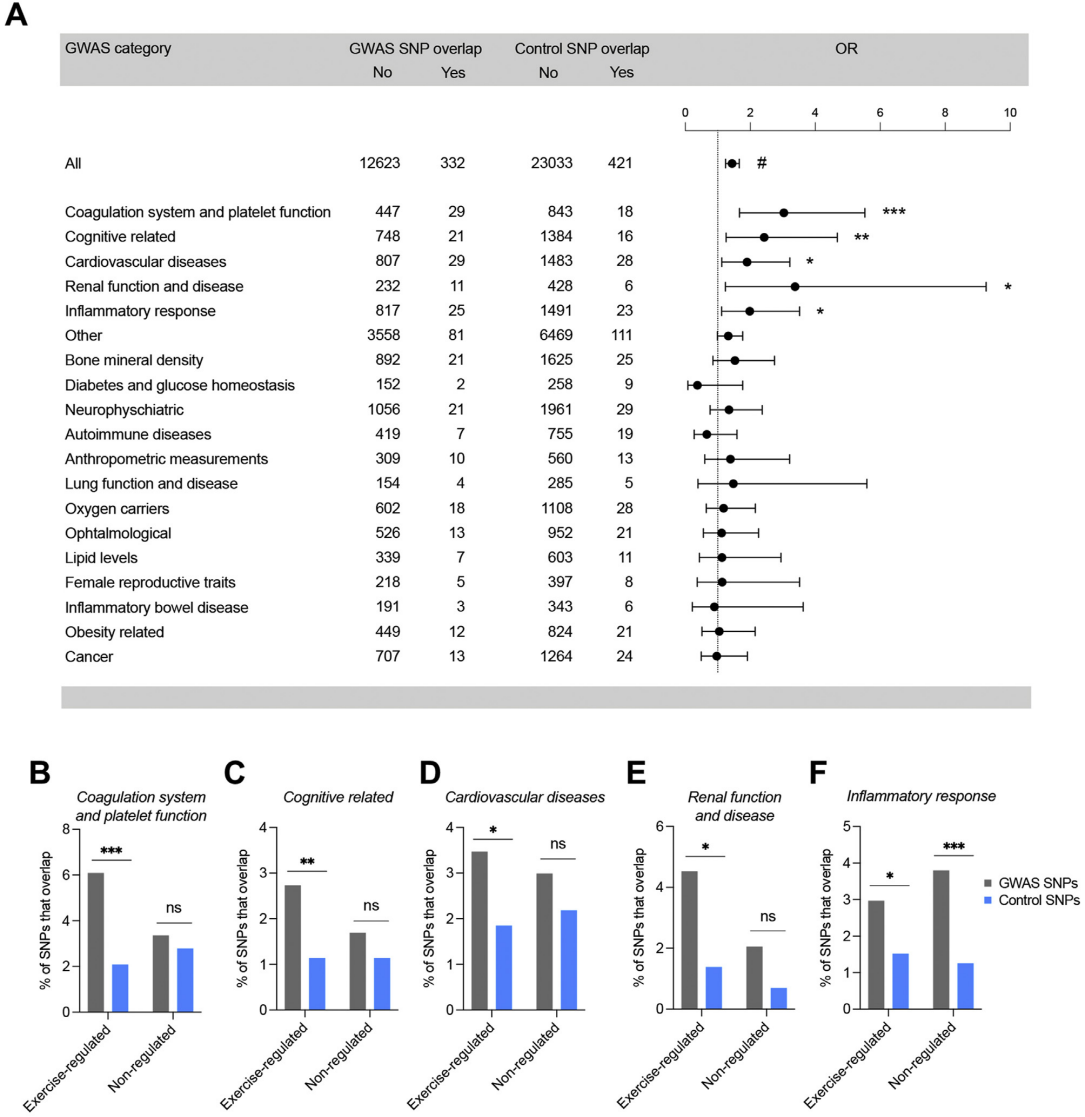
**Enrichment analysis of GWAS SNPs in enhancer regions across different trait categories.** (**A**) Overlap of the control SNPs or GWAS SNPs with training-responsive enhancer regions for all GWAS SNPs together or GWAS SNPs of different disease categories separately. The results are reported as odds ratios (OR) (circles) along with 95% confidence intervals (error bars). ORs were calculated by logistic regression using the overlap between the control SNPs and enhancer regions as the reference value. The dashed line points to OR value of 1. #p < 10^−5^, ∗∗∗p < 0.001, ∗∗p < 0.01, ∗p < 0.05. (**B–F**) The percent of control or GWAS SNPs associated with *coagulation system and platelet function* (B), *cognitive related* (C)*, cardiovascular disease* (D), *renal function and diseases* (E), and *inflammatory response* (F) overlapping with exercise regulated enhancers or nonregulated enhancers.

To gain insight into the role of enhancers regulated by exercise training in whole-body function and human health, we investigated whether GWAS SNPs colocalize with skeletal muscle enhancers that change activity after exercise training. We used a catalog of 12,955 independent GWAS SNPs associated with various diseases and traits and 23,454 matched control SNPs (not associated with any traits), to assess whether GWAS SNPs were enriched in the training-responsive enhancer regions (Supplemental Figure S6). We found that the GWAS SNPs had 1.4-fold higher odds of overlapping with a training-responsive enhancer region than the control SNPs (*p*=7.0 × 10^−6^). More specifically, 332 (2.6%) out of 12,955 GWAS SNPs overlapped a training-responsive enhancer region, whereas 421 (1.8%) out of 23,454 control SNPs showed an overlap (Figure 4A). When we divided the GWAS SNPs into 19 disease categories (Supplemental Figure S7) and assessed the overlap within these subgroups, we found that enhancer regions were enriched for GWAS SNPs associated with the trait categories *coagulation system and platelet function*, *cognitive-related, cardiovascular disease*, *renal function and diseases,* and *inflammatory response* (Figure 4A). To determine if the identified disease categories were specific for exercise-regulated enhancers or just associated with skeletal muscle enhancers in general, we performed a similar analysis using enhancers that were not regulated by exercise. These enhancers were selected by ranking all identified enhancers according to FDR and selecting the 7018 enhancers (the same number as for exercise-regulated enhancers) with the highest FDR (Supplemental Figure S8A). This comparison returned fewer significantly enriched disease categories than the analyses of exercise-regulated enhancers: *inflammatory response, cancer,* and *bone mineral density* (Supplemental Figure S8B). Overall, the results suggest that enhancers regulated by exercise are specifically linked to the modulation of the coagulation system (Figure 4B), to cognitive performance (Figure 4C), cardiovascular disease (Figure 4D), and renal function (Figure 4E); whereas the inflammatory response could be regulated by skeletal muscle enhancers in general (Figure 4F).

Based on the colocalization with GWAS SNPs, we could identify 40 exercise-regulated enhancers that overlap one or more GWAS SNPs and that were connected to a gene that was similarly regulated by exercise training (Supplemental Data S7). We found an overrepresentation of genes predicted to encode for secreted factors, with 24% and 57% reported as secreted factors by Uniprot or ExoCarta, respectively (Supplemental Data S7). Collectively, our findings suggest that enhancers remodeled after exercise training may participate in disease prevention, especially of cardiovascular, renal, and cognitive disorders, by regulating transcription of enhancer connected genes.

## 4 Discussion

Here, we tested the hypothesis that endurance training alters the activity of enhancers in skeletal muscle tissue, which in turn regulates the expression of genes that contribute to the positive effect of exercise on human health. We performed genome-wide enhancer mapping in the human muscle of Caucasian males after exercise training and identified thousands of exercise-remodeled enhancers. By overlapping the enhancer localizations with GWAS data, we further demonstrated that GWAS SNPs are enriched within exercise-regulated enhancers. Our results provide insight into the possible contribution of exercise-induced epigenetic remodeling at enhancer regions on the phenotype.

While changes in gene transcription and protein signaling have been studied extensively during skeletal muscle differentiation and adaptation to a variety of exercise stimuli, only few studies have investigated changes in the enhancer landscape. Enhancer remodeling has been detected during differentiation of mouse C2C12 skeletal muscle myoblasts [57], during muscle cell aging [58], or by the stimulation of human muscle cells with free fatty acids or inflammatory cytokines [59]. Only one study mapped enhancers in skeletal muscle after exercise, which was after four weeks of voluntary wheel running in mice [60]. In concordance with our data, the latter study reported extensive change in the H3K27ac landscape after exercise training, with more enhancers decreasing than gaining acetylation [60]. Strikingly, the top ontologies for genes in proximity to activated enhancers were related to extracellular matrix organization and collagen formation, which further resembles our findings. While most enhancers had decreasing activity after exercise training, the majority of the differentially expressed genes were upregulated. This discordance could be explained by several mechanisms. Firstly, there is not necessarily a linear relationship between genes and enhancers since some genes are controlled more by enhancers than others. Secondly, enhancers can also regulate the expression of noncoding transcripts such as miRNAs and long non coding RNAs, which were not detected in the RNA-seq analysis. Thirdly, the genes and enhancers that were upregulated were generally regulated by a larger fold change than the genes and enhancers that were downregulated. This could indicate that the upregulation of enhancers and genes is the primary effect of exercise training, and the downregulation comes as a secondary effect. Here, the enhancer mapping has less variance than the gene expression analysis. Therefore, we might detect more enhancers that are downregulated, but where the corresponding genes are only slightly downregulated, and hence, not detected in our analysis. Despite this discordance, we still demonstrate an overall correlation between changes in the activity of exercise-remodeled enhancers and expression of connected genes, suggesting that epigenetic remodeling at enhancer regions is likely to drive transcriptional adaptations after endurance training.

Compared to control SNPs with no known disease associations, we found that the locations of SNPs identified in GWAS are enriched in exercise-remodeled enhancers. These findings concur with previous reports showing that the majority of disease-associated SNPs are located in noncoding DNA, notably within enhancer regions [14,61], and reveal the role of cis-regulatory regions in modulating phenotypes. Gene enhancers are highly tissue-specific and amenable to environmental influences [[26], [27], [28]]. By demonstrating the plasticity of skeletal muscle enhancers after exercise training and colocalization with GWAS SNPs, our study provides further evidence that enhancers are functional, and highlights the need for tissue- and context-specific investigations to reveal the function of GWAS SNPs located in non coding regions.

When subdividing GWAS SNPs into disease categories, we found that SNPs associated with *coagulation system and platelet function*, *cognitive performance, cardiovascular disease*, and *renal function and diseases* were enriched in exercise-regulated enhancers and not in non-regulated enhancers. These disease categories are not directly connected to skeletal muscle function, suggesting that remodeling of enhancers in skeletal muscle after endurance training may affect the function of distant organs. Remote action on distant organs could be mediated by the influence of skeletal muscle on the whole-body metabolism, notably, glucose metabolism [62]. However, the fact that many of the identified regulated genes are annotated as *secreted* suggests a regulation of the endocrine – rather than metabolic – function of skeletal muscle tissue in response to exercise training. Skeletal muscle contraction is associated with the release of myokines into the bloodstream [63]. Thus, these protein products of the identified genes may, directly or indirectly, act as endocrine messengers between skeletal muscle and distant tissues after exercise training. However, the GWAS SNPs we found to overlap with exercise-responsive enhancers could potentially control gene regulation in multiple tissues through shared allelic effects or pleiotropy.

The disease categories that we found linked to the reprogrammed enhancers have all previously been associated with exercise. For instance, exercise training is robustly correlated with decreased risk of cardiovascular disease, and more physically active individuals have lower blood pressure and healthier blood lipid profile [64,65]. In addition, physical activity improves cognitive performance [[66], [67], [68]] and associates with higher academic achievement [69,70]. Also, the renal function seems to improve with increased estimated glomerular filtration rate (eGFR) rates and lower blood pressure observed after regular exercise training [71]. In relation to the coagulation system, exercise is associated with increased activation of both coagulation pathways and fibrinolysis [72]. Many of the GWAS SNPs found to overlap with exercise-responsive enhancers are associated with platelet measures, such as mean platelet volume and platelet distribution width [73]. Interestingly, platelet function correlates with a wide range of disorders [74], including cardiovascular risk [75] and neurological disorders [[76], [77], [78]], and platelets are now being recognized for having broader functions than regulating hemostasis. Platelets release proteins and signaling molecules in response to environmental changes and act as transporters of molecules [79,80]. Many of the disease phenotypes that we find connected to exercise-remodeled enhancers could be interconnected through the regulation of platelet functions after exercise. Considering that exercise training reduced the waist-to-hip ratio of the participants, we cannot distinguish whether the effects are directly linked to local adaptations in skeletal muscle or secondary effects from the abdominal weight loss.

We did not identify enrichment of SNPs associated with traits related to blood glucose homeostasis or adiposity. Therefore, we speculate that the effects of exercise training on energy metabolism and blood glucose regulation might be driven through altered intracellular signaling events including regulators like Ca^2+^, AMPK, ROS, and NO, rather than gene regulatory changes in skeletal muscle. In accordance with this, previous GWAS studies have found that most SNPs associated with diabetes affect pancreatic beta-cell function [81], SNPs associated with obesity and BMI primarily regulate genes with functions in the central nervous system [82], and SNPs for waist-to-hip ratio are primarily active in the adipose tissue [83].

In conclusion, our findings that exercise-remodeled enhancers in skeletal muscle are significantly enriched for genetic polymorphisms associated with human complex traits and diseases, suggest the importance of this metabolic organ in the regulation of whole-body phenotypes. By providing insight into the mechanisms that may mediate the positive effects of exercise on cardiovascular function, platelet biology, cognitive performance, and renal function, our study constitutes a powerful resource for the identification of key factors involved in the beneficial effects of endurance training on human health.

### Data statement

All sequencing data have been deposited in the NCBI Gene Expression Omnibus (GEO) and are accessible through GEO Series accession number GSE144134 (https://www.ncbi.nlm.nih.gov/geo/query/acc.cgi?acc=GSE144134).

## Author contributions

K.W., T.O.K., and R.B. conceived the study, planned the experiments, collected all data, and wrote the first version of the manuscript. The final version was read and approved by all authors. I.D. and S.V. recruited human participants and supervised the endurance training intervention. K.W. and M.Y.H. performed the ChIP-seq and RNA-seq experiments. S.H. performed RT-qPCR experiments. N.J.P. *and* J.R.Z. performed correlation analyses between the RNA-seq data and available transcriptomic analyses of exercise. K.W., G.D.C., and L.R.I. conducted the bioinformatic analysis. R.B. is the guarantor of this study.
