# Supplementary material for: Epigenetic rewiring of skeletal muscle enhancers after exercise training supports a role in whole-body function and human health
Source: Mol Metab. 2021 Jul 10;53:101290. doi: 10.1016/j.molmet.2021.101290 (PMC8355925; doi:10.1016/j.molmet.2021.101290)
Supplement: Supplementary file 8 — Multimedia component 8 [file mmc8.docx]

Supplementary Materials for

**Epigenetic rewiring of skeletal muscle enhancers after exercise training supports a role in whole-body function and human health**

Kristine Williams, Germán Darío Carrasquilla, Lars Roed Ingerslev, Mette Yde Hochreuter, Svenja Hansson, Nicolas J. Pillon, Ida Donkin, Soetkin Versteyhe, Juleen R. Zierath, Tuomas Oskari Kilpeläinen, and Romain Barrès^*^.

*Corresponding author. Email: [barres@sund.ku.dk](mailto:barres@sund.ku.dk)

**This PDF file includes:**

Supplemental Figs. S1 to S8

**Other Supplementary Materials for this manuscript include the following:**

Supplemental Data S1 to S7

**
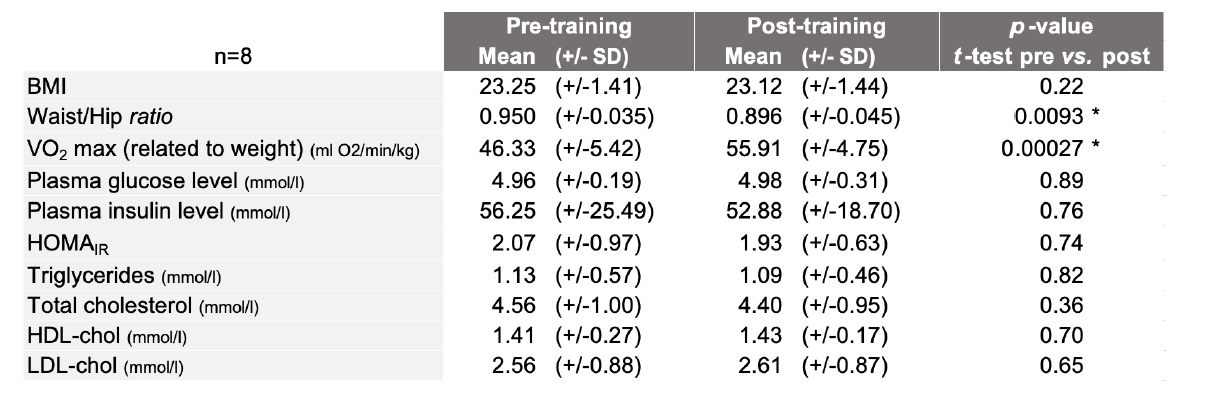
**

**Supplemental Fig. S1.**

Clinical parameters of participants (n=8) measured pre- or post-training. Statistical testing was performed by a two-sided paired t-test.

**
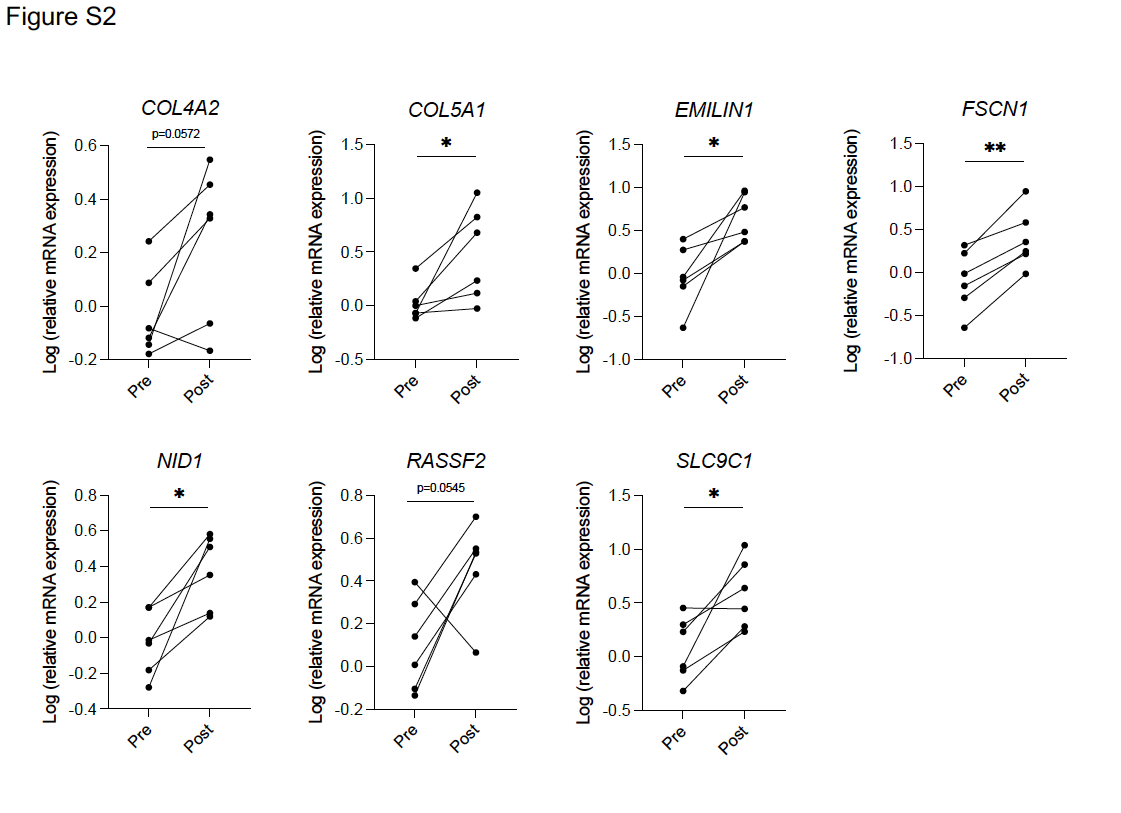
**

**Supplemental Fig. S2.**

Real-time quantitative PCR of genes that were found upregulated by exercise training in the RNA-seq analysis (n=6 participants, student t-test, * p<0.05).

**
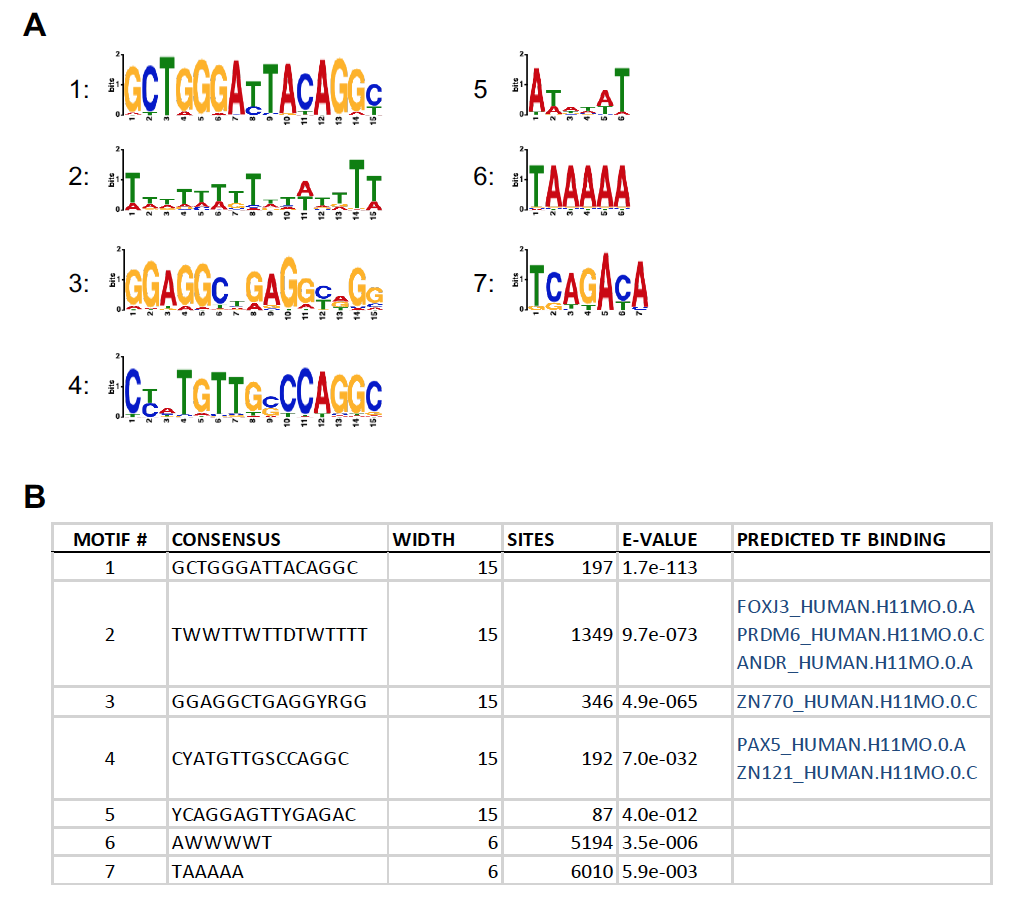
**

**Supplemental Fig. S3.**

**A-B**, Motifs that were found enriched within exercise regulated enhancers (A) and their corresponding width, number of sites found, the FDR-value (E-value) and which transcription factors (TFs) that are predicted to bind the given motif (B).

**
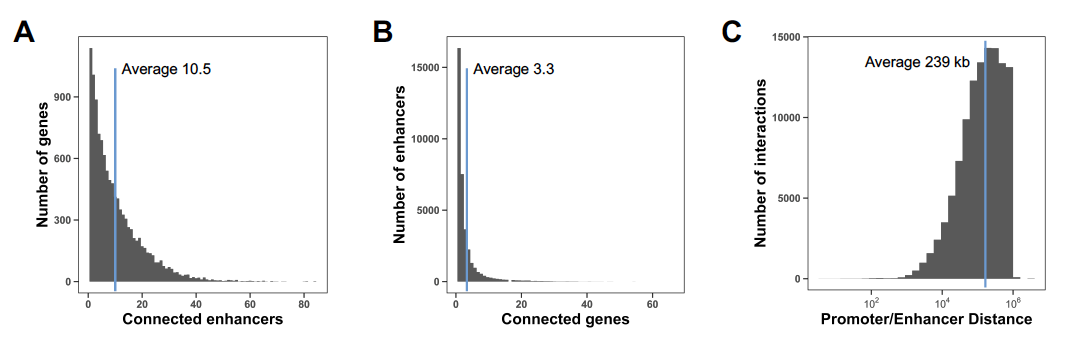
**

**Supplemental Fig. S4.**

**A**, Histogram displaying the number of enhancer interactions per gene. The average number of interactions is 10.5. **B**, Histogram displaying the number of gene interactions per enhancer. The average number of interactions is 3.3. **C**, Histogram displaying the distances between interacting promoter- and enhancer-fragments. The average distance is 239 kb.

**
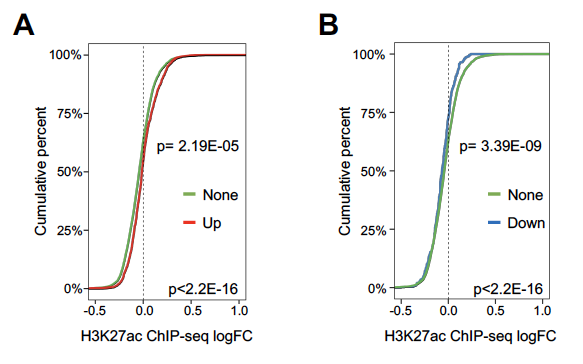
**

**Supplemental Fig. S5.**

**A-B**, Enhancers were divided into three groups; enhancers connected to genes that did not change expression in response to exercise training (“None”), and enhancers connected to genes that either gained expression (“Up”) or lost expression (“Down”) in response to exercise training. The figures illustrate empirical cumulative distribution function (EDCF) plots of enhancer H3K27ac changes in the “Up” versus the “None” group (A) or the “Down” versus the “None” group (B).

**
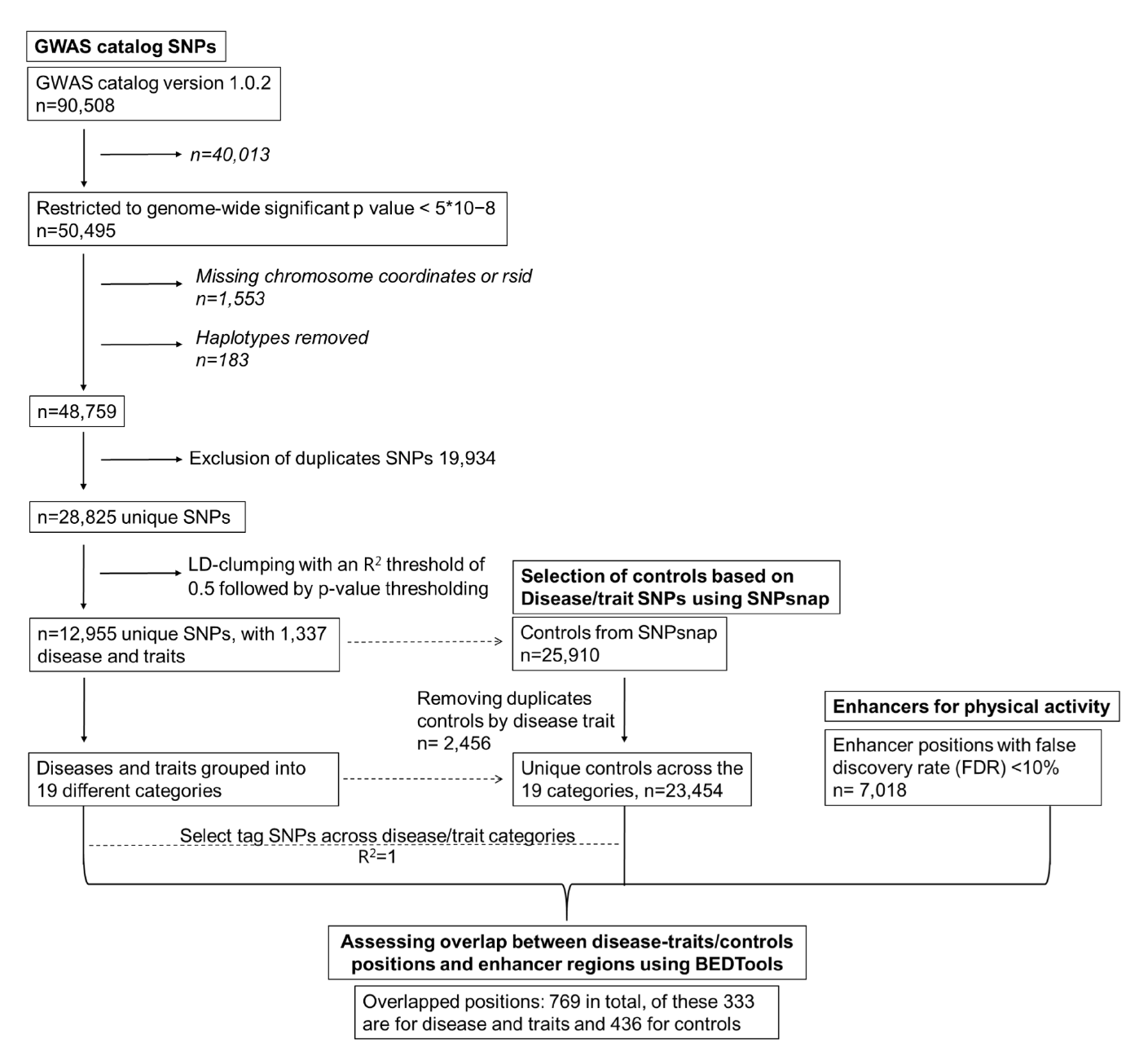
**

**Supplemental Fig. S6.**

Schematic overview of data retrieval and analyses associated with the enrichment analysis of GWAS SNPs in enhancer regions.

**
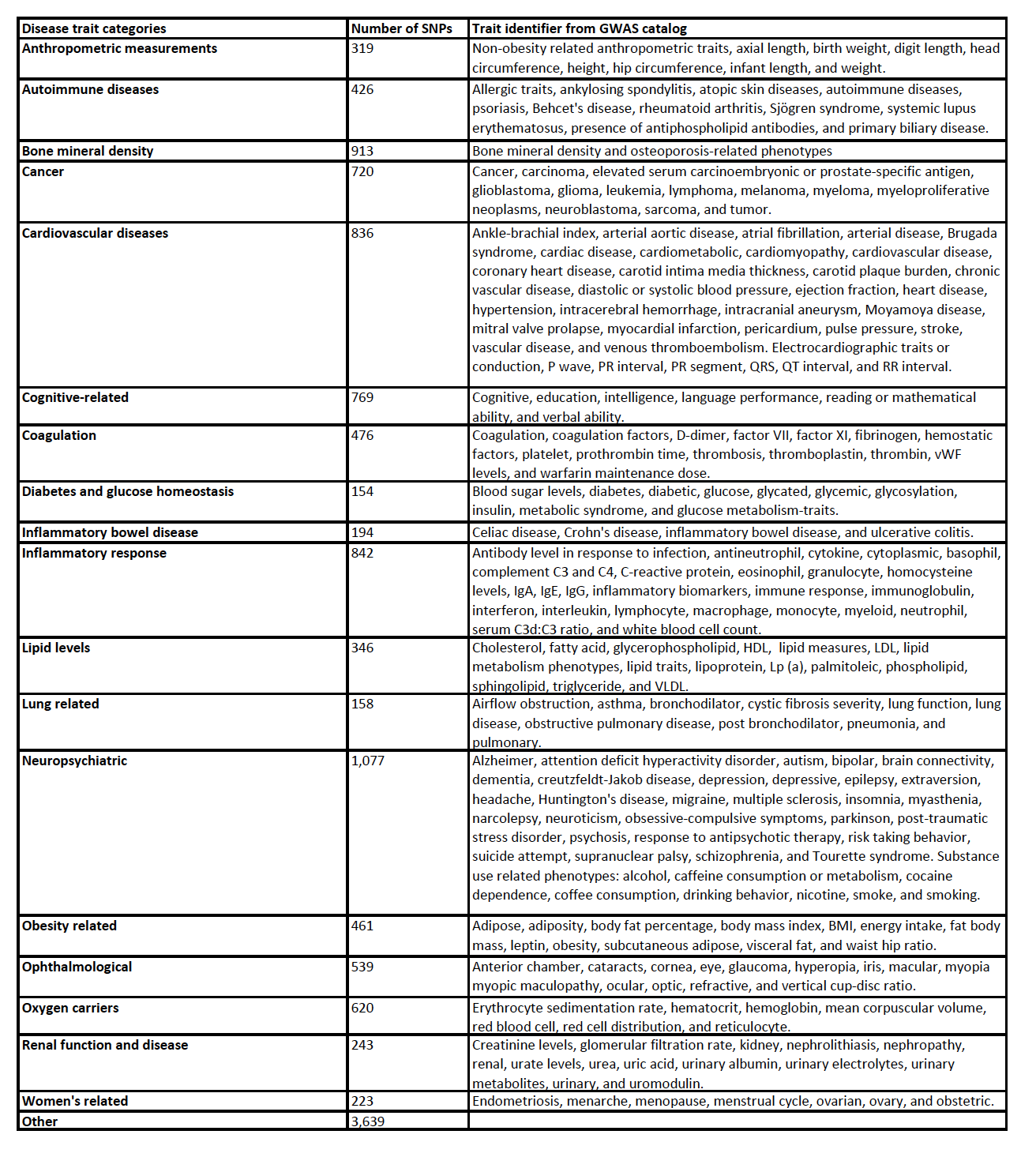
**

**Supplemental Fig. S7.**

Overview of grouped categories of GWAS traits.

**
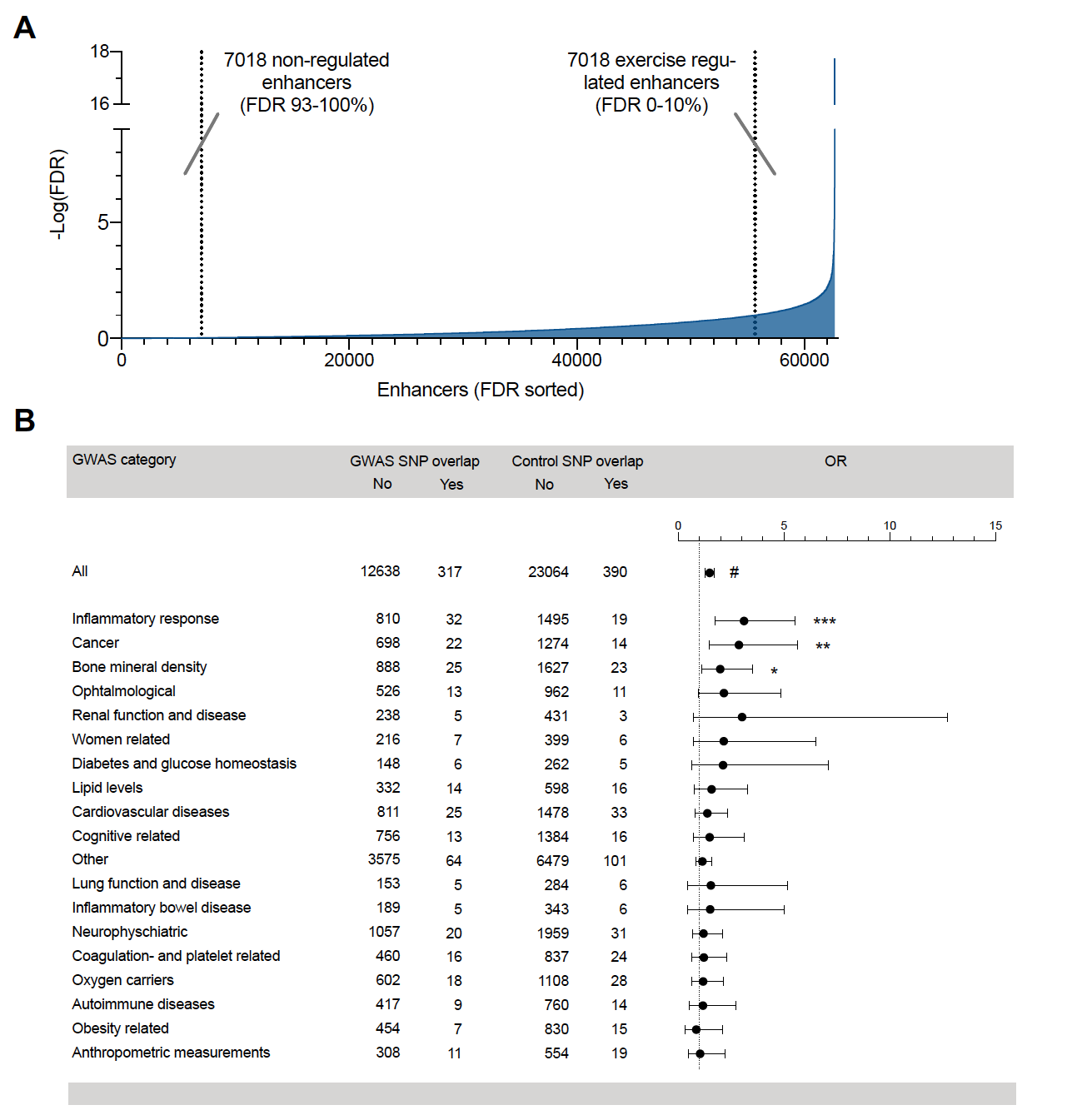
**

**Supplemental Fig. S8.**

**A,** Enhancers were ranked according to FDR values and the overlap with GWAS and control SNPs was tested for the 7018 enhancers with an FDR value of 0-0.1 (exercise-regulated enhancers). For comparison, a similar analysis was performed for the 7018 enhancers with the highest FDR (non-regulated enhancers). **B,** Overlap between the control SNPs or GWAS SNPs with non-regulated enhancer regions for all GWAS SNPs together or GWAS SNPs of different disease categories separately. Results are reported as odds ratios (OR) (circles) along with 95% confidence intervals (error bars). ORs were calculated by logistic regression using the overlap between the control SNPs and enhancer regions as the reference value. The dashed line points to OR value of 1. #p<10^-5^, ***p<0.001, **p<0.01, *p<0.05.

**Supplemental Data S1 (separate file).**

Differentially expressed genes. “LnFC” indicates the fold change in gene expression levels between pre- and post-training skeletal muscle samples.

**Supplemental Data S2 (separate file).**

Gene ontologies of differentially expressed genes.

**Supplemental Data S3 (separate file).**

List of genes that were regulated in the RNA-seq analysis and that encodes a gene product annotated as secreted by Uniprot or ExoCarta.

**Supplemental Data S4 (separate file)**.

Genomic position (hg38) of all identified enhancer regions (H3K4me1 and H3K27ac positive). “LogFC” indicates the fold change in H3K27ac levels between pre- and post-training skeletal muscle samples.

**Supplemental Data S5 (separate file).**

Ontology analysis of genes close to exercise-regulated enhancers.

**Supplemental Data S6 (separate file).**

List of all enhancers that were found connected to a gene in skeletal muscle, and where both the enhancer and the gene was changed after exercise training (in same direction).

**Supplemental Data S7 (separate file).**

List of all enhancers overlapping one or more GWAS SNPs that were found connected to a gene in skeletal muscle, and where both the enhancer and the gene was changed after exercise training (in same direction).
